# Mature cystic teratoma of the neck misdiagnosed at cystic hygroma; Case report

**DOI:** 10.1016/j.ijscr.2020.12.062

**Published:** 2020-12-24

**Authors:** Hassan Muhsen Hassan, Asaad Shareef Omar, Ayad Ahmad Mohammed

**Affiliations:** Department of Surgery, College of Medicine, University of Duhok, Kurdistan Region, Iraq

**Keywords:** Teratoma, Cystic teratomas, Maure cystic teratoma, Cystic hygroma, Neck mass, Toti-potential germ cells

## Abstract

**Background:**

Teratomas arise from primordial germ cells which arrest during its migration from the hindgut allantois the gonads during the first weeks of gestational life, they may occur in both gonadal and extra-gonadal locations. They occur in 1/40,000 live births. The most common anatomical locations are the sacro-coccygeal region and the ovary, neck teratomas constituted about 1.5%. Malignant transformation has been reported.

**Case presentation:**

A 2-year-old boy presented with a gradually enlarging mass in the left side of the neck causing stridor and difficulties in respiration especially during sleep, the parents noticed difficulties during swallowing. The mass was misdiagnosed as cystic hygroma and the patient underwent 2 sessions of sclerotherapy with no improvement. Clinical examination showed a large mass in the left side of the neck which was multilobulated causing tracheal shift to the opposite side. There were no signs of inflammations over the mass. CT-scan showed evidence of enhancing multi-cystic lesion with multiple flecks of calcification. Complete surgical resection was done successfully and the histopathological study of the mass showed mature elements of ectodermal, mesodermal, and ectodermal germinal layers which was consistent with mature cystic teratoma. The follow up showed no postoperative complications.

**Conclusion:**

Mature cystic teratoma of the neck is very rare and may be difficult to be differentiated both clinically and radiologically from other neck masses. The accurate diagnosis must be made before any kind of treatment is started. Complete surgical resection is the main management option and required to decrease the recurrence.

## Highlights

• The incidence of teratomas is approximately 1/40,000 live births.
• Histologically, they are heterogeneous with mixed cystic, solid, mature, and immature components.
• Most head and neck teratomas are small and amenable to curative surgical resection.
• Neck teratomas are usually misdiagnosed initially.

## 1 Introduction

Teratoma is an embryonal neoplasms that originate when the toti-potential germ cells escape the normal development and change to tumors that contain various types of tissues which derive from all three blasto-dermic layers [1].

Embryonically, teratomas arise from primordial germ cells which arrest during its migration from the hindgut allantois the gonads during the first weeks of gestational life. Due to this migration they may occur in both gonadal and extra-gonadal locations and can be localized anywhere from the head to the coccyx [[1], [2], [3]].

The incidence of teratomas is approximately 1/40,000 live births with slight female predominance. The most common anatomical locations of teratomas are the sacro-coccygeal region and the ovary, rarely they may occur in other sites like the mediastinum and any other anatomical site, neck teratomas constituted about 1.5% of all teratomas [1,3].

Histological examination show that teratomas are composed of heterogeneous with mixed cystic, solid, mature, and immature components. Histologically they are classified into mature teratomas which are benign and constituted about 95% of cases and immature teratomas which involve malignant transformation [1].

Teratomas are usually asymptomatic and clinically vary greatly in size, they may be small in size but sometimes they may reach an enormous size causing pressure effects resulting in obstruction of the respiratory or digestive passages [1,3].

Imaging help to detect the size and the extension of the mass, ultrasound show the size and the composition and detect any cystic components inside the mass, CT scan and MRI are more useful to show the anatomical details and the origin of the mass [1].

Surgery is the main management option and complete surgical resection required to decrease the rate of recurrence, malignant transformation has been reported in some cases and such cases require more extensive surgical resection. Radiotherapy and chemotherapy are usually not very effective in such cases [1,3].

The aim of presenting this case is to highlight the differential diagnosis of neck masses in children and the role of surgery and histopathology in the confirmation of teratomas that may be misdiagnosed and managed as cystic hygroma.

The work of this report case has been reported in line with the SCARE 2020 criteria [4].

## 2 Patient information

A 2-year-old boy presented with a gradually enlarging mass in the left side of the neck causing stridor and difficulties in respiration especially during sleep, the parents noticed difficulties during swallowing. There were negative personal or family histories for medical or other genetic disorders. The drug history was also negative.

The mass was misdiagnosed as cystic hygroma of the neck and the patient underwent 2 sessions of sclerotherapy with no any clinical improvement.

### 2.1 Clinical findings

**Fig. 1:**
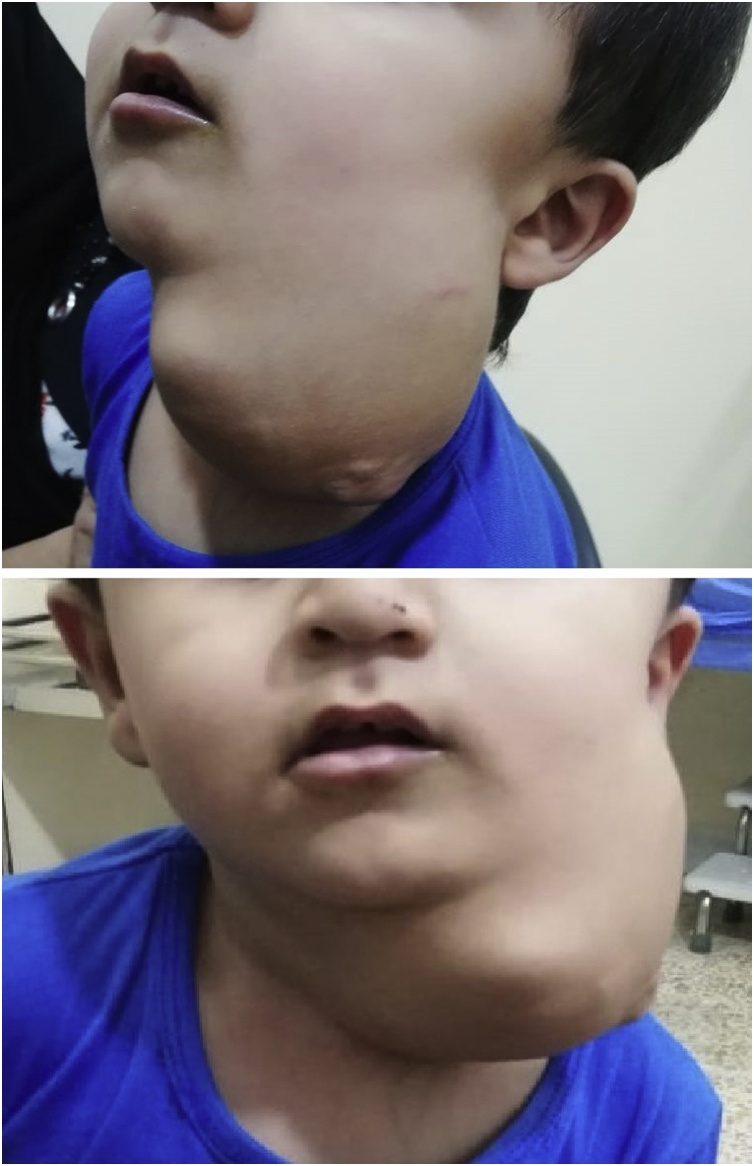
A preoperative picture showing the multilobulated mass in the left side of the neck.

During clinical examination the patient had a large mass in the left side of the neck, its size was 10 × 8 × 8 cm in maximum dimensions. The mass was extending to the left check superiorly, the posterior neck, the supraclavicular region inferiorly, and crossing the midline of the anterior neck. The mass was multilobulated with areas mixed soft and solid consistencies during palpation. The trans-illumination test was negative. The trachea was shifted to the opposite side. There were no signs of inflammations over the mass. The vital signs were normal and other parts of the examination were unremarkable (Fig. 1).

### 2.2 Diagnostic assessment

**Fig. 2:**
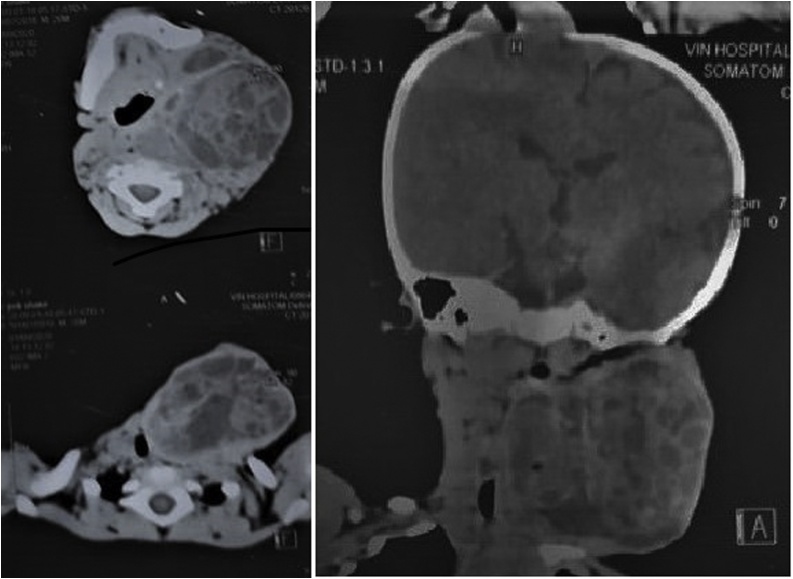
CT-scan of the head and neck showing an evidence of enhancing multi-cystic lesion measuring 10 × 8 × 8 cm with multiple flecks of calcification. The mass is extending to the mastoid bone and the clavicle and causing displacement of the trachea, esophagus, and carotid sheath to the opposite side.

CT-scan showed evidence of enhancing multi-cystic lesion with multiple flecks of calcification and causing displacement of the trachea, esophagus, and carotid sheath to the opposite side. There were no enlarged cervical lymph nodes (Fig. 2).

### 2.3 Therapeutic intervention

**Fig. 3:**
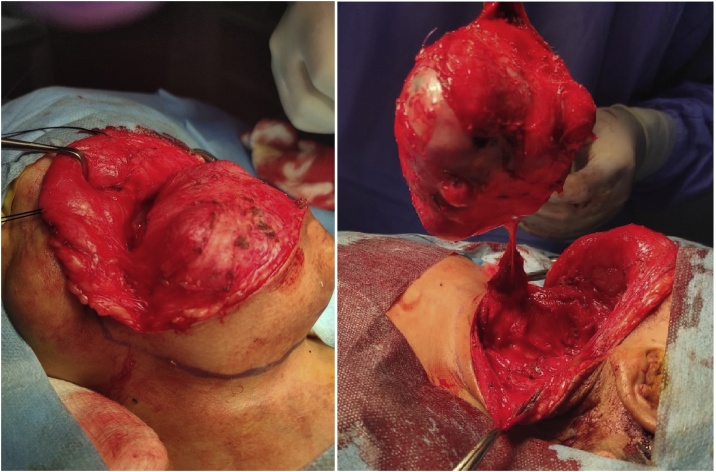
An intraoperative picture showing the neck mass with complete dissection from the neck structures.

**Fig. 4:**
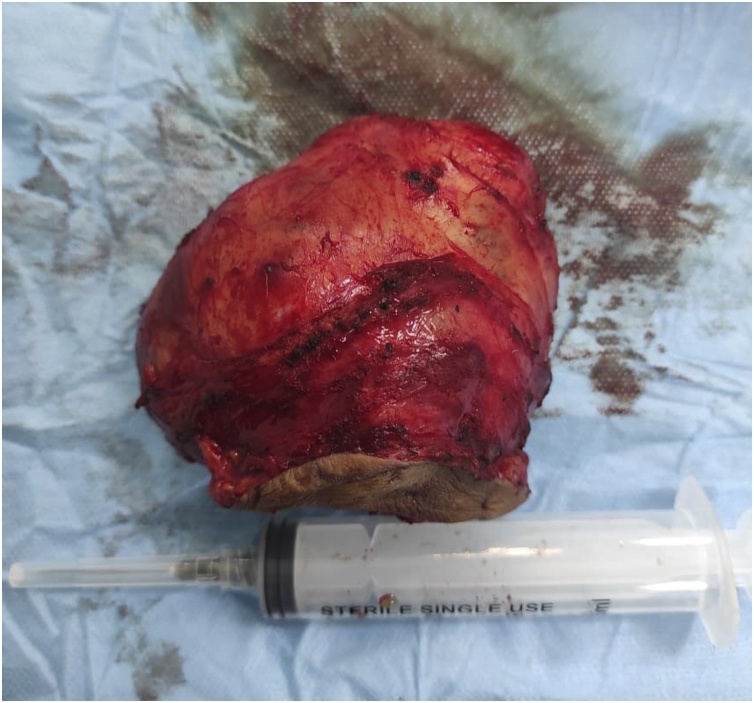
An intraoperative mass showing the mass after being completely excised.

The patient was admitted for surgical intervention. An oblique elliptical was done over the mass, dissection done and the mass was completely separated from the neck structures and complete surgical excision was performed. There were no signs of local invasion (Fig. 3, Fig. 4).

The operation was done by 2 general surgeons who are specialized in the field of general surgery and neck surgery.

**Fig. 5:**
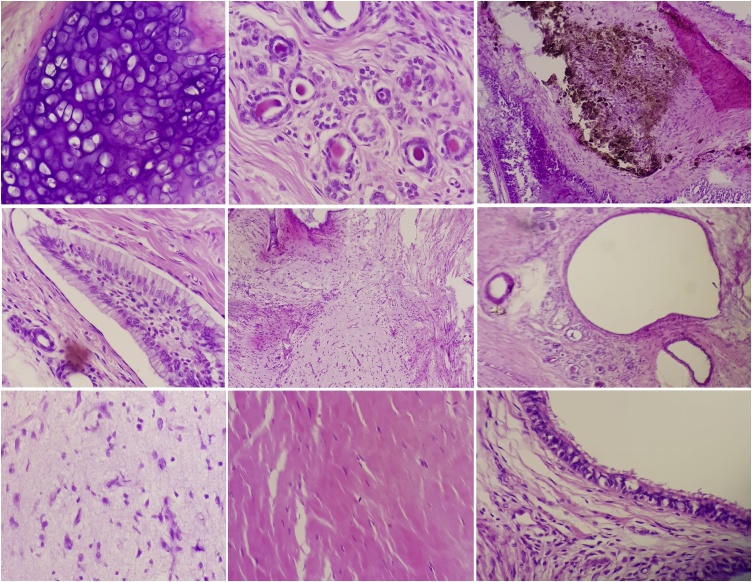
Microscopic pictures showing the different mature elements inside the mass; there is evidence of cartilaginous tissue, thyroid tissue, melanocytes, intestinal epithelia, respiratory epithelia, neural tissue and skeletal muscle tissues.

The histopathological study of the mass showed mature elements of ectodermal, mesodermal, and ectodermal germinal layers. No immature elements were seen. The appearance was consistent with mature cystic teratoma (Fig. 5).

### 2.4 Follow-up and outcomes

The patient was admitted for 3 days after surgery, the drain was removed at the 2nd postoperative day. The follow up showed no postoperative complications. No specific postoperative considerations were present.

## 3 Discussion

Teratomas are composed of all the three germ cell layers: ectoderm, mesoderm and endoderm that exhibit various degrees of maturity, typically they consist of mature elements of various types of tissues. Teratomas can occur in gonadal or extra-gonadal locations. Sacrococcygeal teratomas are most common in children and teratomas of the gonads are most common in adults, neck teratomas are relatively rare and usually are found as an extension from mediastinal origin [1,2,5,6].

They present as slowly enlarging masses unless if they developed malignant transformation, classically they exhibit solid or mixed cystic and solid components. The majority of mature teratomas contain epithelial and sebaceous components [2].

Cystic teratomas may be difficult to be differentiated both clinically and radiologically from lymphatic vascular malformations and such patients may be treated with sclerotherapy which usually shows no response [7].

Most teratomas contain fat which is of mesodermal origin and it is an imaging hallmark and has a very important diagnostic value during imaging. The contents of the teratomas are best assessed by CT scan. CT scan frequently assess the presence of coarse or fine linear patterns of calcium deposition. The detection of fat inside the tumor whether as sebum or macroscopic fat is the most characteristic finding of mature teratomas. Fat is demonstrated in standard imaging and typically has less than −20 HU attenuation on CT scan images and a decreased signal intensity on fat saturated MRI images [2,8].

Neck teratomas must be differentiated from cystic hygroma, branchial cyst, hydatid cyst, enlarged cervical lymph node, and thyroid related pathologies. When a child present with one congenital malformation, complete clinical examination should be done to role out other possible associated malformations [1,[9], [10], [11], [12]].

Some complications have been reported in patents with mature teratomas such as infection, rupture, malignant transformation, hormone secretion which occur particularly in cases of struma ovarii, torsion in cases of ovarian teratomas. Malignant transformation is estimated to occur in 2%–3% and is more frequent in children and can originate from any of the three germinal cell layer, the majority of malignant transformations give rise to squamous cell carcinoma, and other types of cancers may include adenocarcinoma and sarcoma. Malignant transformation is suspected when there is rapid change in size, abnormal wall thickening and irregular walls, and evidence of local invasion [2].

Most head and neck teratomas are small and amenable to curative surgical resection, sometimes patients may have delay presentation and they may reach a very large size making surgical intervention challenging. In adult patients, immature tumors are more likely to exhibit malignant behavior [8,13,14].

The prognosis is usually favorable providing that the correct diagnosis is made. In a case series presentation involving 23 patients with head and neck teratomas, the authors reported an excellent prognosis after surgical intervention. The presented case had been treated successfully with surgery and the follow up showed no postoperative complications and recurrence [15,16].

Neck masses may be misdiagnosed due to the variety of differential diagnoses, our particular case was misdiagnosed as cystic hygroma and because of no response to sclerotherapy we thought of an alternative diagnosis.

## Declaration of Competing Interest

The author has no conflicts of interest to declare.

## Funding

None.

## Ethical approval

Ethical approval has been exempted by my institution for reporting this case.

## Consent

Written informed consent was obtained from the patient for publication of this case report and accompanying images. A copy of the written consent is available for review by the Editor-in-Chief of this journal on request.

## Author contribution

Dr Hassan Muhsen Hassan and Dr Asaad Shareef Omar contributed to the concept of reporting the case and the patient data recording.

Drafting the work, design, and revision done by Dr Ayad Ahmad Mohammed.

Final approval of the work to be published was done by Dr Ayad Ahmad Mohammed.

## Registration of research studies

This work is case report and there is no need of registration.

## Guarantor

Dr Ayad Ahmad Mohammed is guarantor for the work.

## Provenance and peer review

Not commissioned, externally peer reviewed.

## Patient perspective

The parents were hoping a complete cure for their child, after surgery and the result of histopathology they were very hopeful and happy about the results of both surgery and the final diagnosis.
